# Assessment of online pharmacy applications in India by employing the mobile application rating scale

**DOI:** 10.3389/fpubh.2024.1486990

**Published:** 2024-11-13

**Authors:** Anum Sattar, Safila Naveed, Hina Rehman, Shahnaz Usman, Shazia Jamshed

**Affiliations:** ^1^Faculty of Pharmacy, Jinnah University for Women, Karachi, Pakistan; ^2^Department of Pharmacy Practice, Faculty of Pharmacy, Ziauddin University, Karachi, Pakistan; ^3^Department of Pharmaceutical Chemistry, Karachi University, Karachi, Pakistan; ^4^Department of Pharmacy Practice, Institute of Pharmaceutical Sciences, Jinnah Sindh Medical University, Karachi, Pakistan; ^5^Department of Pharmaceutics, RAK Medical & Health Sciences University, Ras Al Khaimah, United Arab Emirates; ^6^Department of Pharmacy Practice, School of Pharmacy, International Medical University (IMU), Kuala Lumpur, Malaysia

**Keywords:** India, MARS, online, pharmacies, mobile applications

## Abstract

**Introduction:**

Over the past few years, the technology powering mobile devices such as smartphones has made significant progress. Furthermore, the healthcare industry is always progressing and actively embracing the latest technological advancements to achieve the highest level of efficiency. With the rising prevalence of smartphones and internet connection, customers are benefiting from reduced prices, convenient home delivery, and effortless accessibility through online pharmacies. Internet-based pharmacies facilitate the internet-based transaction of health-related products, such as drugs, dietary supplements, and various other wellbeing products.

**Objective of study:**

The study assessed digital pharmacy applications in India using the Mobile App Rating Scale (MARS) on Android and iOS devices, aiming to evaluate their quality.

**Methods:**

An investigation examined the digital pharmacy applications in India that were accessible via the Android Market and App Store. The applications were assessed by two researchers using the MARS questionnaire, a tool that evaluates 23 variables categorized into five domains: Engagement, Functionality, Aesthetics, Information, and Subjective Quality. The grading system spanned from one to five for every category.

**Results:**

A Google Play Store and App Store investigation revealed 40 online pharmacy apps in India, with 13 rejected. Seven were non-English language-related apps and seven were not downloaded. Thirteen were chosen and evaluated using the MARS Scale. The MARS demonstrated significant positive associations across its components, namely Engagement, Functionality, Aesthetics, and Information. Specifically, greater levels of user functionality were shown to be indicative of superior app aesthetics and engagement. The mean rating of the 13 apps fell between the range of 3.11 to 4.32 on a 5-point scale.

**Conclusion:**

This is the first study to utilize the MARS scale to assess the efficacy of online pharmacy applications in India. This research enhanced the functionality and quality of various online pharmacy applications utilized in India.

## 1 Introduction

Online pharmacy is an internet-based platform or website that provides medicines, encompassing both prescription and non-prescription medications, along with a range of goods such as healthcare and cosmetic items (1). Many online pharmacies deliver orders with an approved prescription, others offer virtual consultations for prescribing and administering medications, whereas others supply meds without a doctor's order. Online pharmacy applications enable patients with rapid access to purchase medications from home, enhancing adherence via prescription refills and health information (2). In modern culture, people are increasingly dependent on the Internet for staying informed about health-related issues and for self-diagnosing and obtaining a wide variety of healthcare services and merchandise (3). The explosive rise of the World Wide Web, the emergence of digital health, a decrease in conventional doctor-patient interactions, customer comfort via purchasing goods online, the ease of mail-order commerce, and the development of separate selling are some of the factors that have contributed to the increase in customer appetite for electronic pharmacies (4, 5). The online pharmacy sector has seen significant growth in the past few years, with its total market value rising from $29.35 billion in 2014 to a projected $128 billion by 2023. The mentioned increase corresponds to a yearly rate of expansion of 17.7% (6).

Online advertising is now in its early stages of adoption in the Indian pharmaceutical business. Pharmaceutical businesses are using technology-based services to enhance patient education about their medical problems and to allow monitoring of their health. Furthermore, it is crucial to provide physicians with precise information on the patient's general health condition and any possible adverse effects of a certain chemical. Online tools can enable improved doctor-patient engagement based on particular health issues (7). Presently, there is a significant global presence of ~3,000 internet pharmacies. Indian customers are progressively demonstrating an escalating preference and shift toward online pharmacies, with this pattern gaining impetus over time. The expansion of the internet-based pharmaceutical industry in the Indian market may be ascribed to the increasing levels of internet awareness, heightened smartphone use, and the availability of efficient transaction options. The online pharmacy industry has lately become a significant player in the Indian e-commerce market, garnering interest from both government entities and business owners. The online pharmacy industry in India has achieved a value of over one billion dollars, thanks to the backing of 30 recently founded start-ups functioning in different sectors and regions of the nation (8).

In the year 2019, India had an estimated 850,000 pharmacy stores, accounting for 99% of the country's pharmaceuticals and medication sales. Conversely, internet-based pharmacies constituted a mere 1% of the overall medicine sales in India (9). In 2018, the number of internet customers in India reached ~560 million (56 crores), with a corresponding number of installations of 12.3 billion. This figure positions India as the second largest in the world, trailing only China. The cumulative duration of social media usage among individuals in India amounts to 17 h over 7 days, surpassing the average time spent by social media users in both China and the United States (10). India's pharmaceutical business holds the position of being the third-largest industry worldwide (1). Many individuals are inclined to adhere to prevailing patterns, and presently, the prevailing pattern is to engage in internet shopping due to its widespread convenience. Drugstore.com was established in 1999. Drugstore.com cannot be considered the pioneering online pharmacy, yet it is widely acknowledged as a reputable and reliable entity within the realm of online pharmaceutical services.

The Indian online pharmacy sector had significant market growth in 2015 (7, 11). In 2015, 11 online or internet pharmacy start-ups came together to establish the Indian Pharmacy Association (IPA). The organization comprises many members, namely MG, Net Meds, Bookmeds, mChemist, Medlife, Pharmacy, Medidart, Medstar, Zigy, Save on Medicals, and Save My Medicines (12).

The idea of an Internet pharmacy differs significantly from regular e-commerce trading due to the importance of adhering to distinct norms and regulations. There is a possibility of jeopardizing individuals' safety and wellbeing If health-related applications and devices are not submitted for comprehensive evaluations of their appropriateness and reliability. Multiple research projects have underscored the issue of unreliability in corroborating information within medical applications. The growing apprehension about the potential hazards associated with the use of medical applications has resulted in a heightened interest in monitoring the efficacy and reliability of these electronic devices. Over the previous 10 years, a team of researchers, under the leadership of Stoyan et al., devised the MARS (13). The MARS is a widely recognized and reliable assessment tool specifically developed for evaluating the functionalities of mobile health (mHealth) applications (13, 14). The technique consistently demonstrates strong dependability and provides a wide range of potential applications when used to comprehensively analyze the material of mobile applications. The MARS model consists of 23 distinct components that are categorized into multiple domains, such as involvement, utility, layout, data correctness, and subjective assessment (14–25).

## 2 Objective of study

The objective of our study was to perform a thorough assessment of digital pharmacy applications in India that were accessible on the Play Store on Google's Android devices and the App Store for iOS devices. To attain the above-indicated objective, a thorough assessment of these applications was carried out by implementing the MARS.

## 3 Methodology

### 3.1 Information on assets and investigation techniques

Two assessors, who specialized in pharmacy and have received education in the field of MARS, conducted a comprehensive search for digital pharmacy applications on the official marketplaces of the two leading operating systems, namely Android (Play Store) and iOS (App Store). To ensure the inclusiveness of the results, a highly popular search term or search string specifically “online pharmacy apps in India,” or Indian pharmacy, Indian medicine store, was employed to identify the relevant applications.

### 3.2 Parameters for acceptance

The sample selection criteria for the study were determined by a combination of inclusion and exclusion criteria. The inclusion criteria encompassed programs that were exclusively developed in India, available for download from both the Google Play Store and Apple iTunes Store, and relevant to the domain of online pharmacies. The study did not include biases linked to financial or premium apps and only apps in the English language were included in the analysis. The requirements for exclusion consisted of the omission of apps that required payment, apps that were not in the English language, apps that were not available for installation on Google Play and Apple iTunes stores, and any duplicates.

### 3.3 The features and attributes connected with applications

The compilation of the entire dataset entailed the incorporation of essential data derived by the applications. The data collection procedure involved gathering various properties of the applications, including the application's name, device compatibility (Android or iOS), cost, category (related to healthcare, wellness, and activity), date of the most recent update, primary language, number of reviews, rating, developer information, and download count.

### 3.4 The selection technique for online applications

The two independent evaluators conducted assessments on the titles and downloading apps from platforms. The applications that exhibited potential applicability were recorded in a database, while any ambiguities led to their exclusion. The applications that met the predetermined criteria were retained, while those that did not satisfy the specified conditions were excluded. When there is doubt over the appropriateness of an application, a third evaluator is involved.

### 3.5 Collecting and evaluating the integrity of information

The two evaluators who were pharmacists and received education on the MARS tool by watching a tutorial video available on the online site, YouTube (https://www.youtube.com/watch?v=25vBwJQIOcE). Following this, the evaluators proceeded to autonomously download, utilize, and evaluate the other software, also known as apps. To collect data, the investigators applied a comprehensive data collection form that included various pertinent details. These details included information about the application's designer, the device on which it was used, the current version of the application, the year of its latest release, associated costs, the number of downloaded files, star rating from consumers, the presence of a guarantee of security, privacy-related technical variables, medical device data, and the components evaluated through the Mobile App Rating Scale.

### 3.6 Investigative instrument

The evaluation of incorporated applications' usability utilized the Mobile App Rating Scale, a comprehensive tool comprising 23 criteria categorized into five groups. The engagement (A) assessment covers factors such as enjoyment, user interest, personalization, interaction (including alerts, messages, signals, and comments), and suitability for the target demographic.

The functionality (B) of the application includes 4 key aspects: operational efficiency, ease of utilization, directions, conceptual flow, and gestures layout. The aesthetics section (C) assesses graphic layout elements, visual attractiveness, chromatic palette, and aesthetic coherence. Another part Information (D) examines the existence of quality data, including textual content, comments, evaluations, and citations from reliable sources.

The subjective quality section (E) consisting of four items, evaluates the consumer's level of enthusiasm for the application on a rating scale from one (indicating inadequacy) to five (representing excellence). The composite app grade was determined by computing the average score across parts A, B, C, and D. Application performance results, a subjective measure, was derived separately by calculating the mean value of the subsection. Subsection (F) perceived impact included six app-specific questions assessing the perceived impact of the application on users' understanding, attitudes, intentions for change, and the likelihood of successful behavior modification related to the targeted wellness behavior.

To enhance the objectivity of the MARS scale in evaluating application excellence, the subjective aspect of the quality part was excluded from the overall average app performance grade calculation. Moreover, the strong association demonstrated in prior research between the MARS sum score and individual star ratings suggests its effectiveness in reflecting overall perceived excellence (13).

### 3.7 Compiling and evaluating data

Each reviewer autonomously assessed all elements within the MARS for each of the applications. The numerical evaluations provided by both evaluators were used to calculate the average rating for each item within each app. Subsequently, the average scores were aggregated to analyze the overall mean scores for each category and section of the MARS across all applications. Mean scores and standard deviations were then computed for each area, applying the same methodology to derive the overall score and its corresponding standard deviation for each application.

### 3.8 Statistical analysis

The data underwent analysis using SPSS version 27. The examination of every facet of MARS involved utilizing the mean value, following the recommendation of its creators. Mean, standard deviation, and Pearson correlation analyses were employed for all dimensions of MARS. To evaluate the internal reliability of the MARS quality subscales, Cronbach's alpha was applied. This statistical metric gauges the degree of associations among components assessing the same underlying construct and their ability to yield comparable results. The intraclass correlation coefficient (ICC) was utilized to assess the internal consistency of the MARS subscales.

## 4 Results

**Figure 1:**
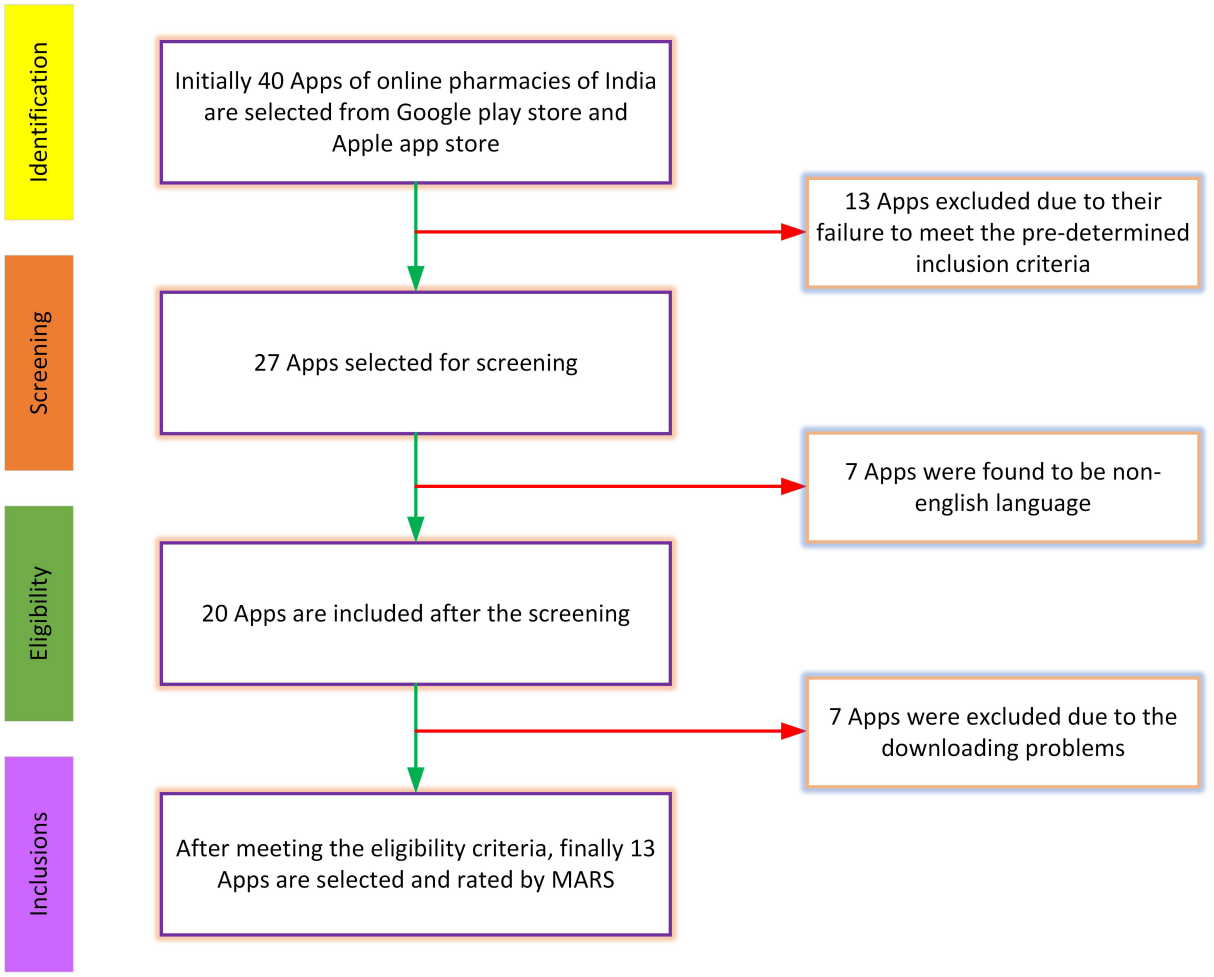
Flowchart outlining the process for selecting applications.

We initiated a preliminary search on both the Google Play Store and the App Store to identify 40 online pharmacy applications available in India, as illustrated in Figure 1. Thirteen apps were excluded from the study as they did not meet the inclusion criteria. Additionally, seven applications were omitted from the analysis because they were based on non-English languages. Furthermore, seven applications were not downloaded for this study. The final selection for inclusion in the study, as depicted in Figure 1, comprised a total of 13 applications.

### 4.1 Attributes of the applications

Mobile applications have become an essential aspect of contemporary life, particularly within the healthcare and pharmacy sectors. These apps provide convenient access to a diverse range of healthcare and pharmaceutical products and services. The tables below offer a comprehensive analysis of healthcare and pharmacy-related applications in India, covering technical features, creator details, customer reviews, pricing, version specifications, affiliations, and other relevant information. Table 1 provides a detailed overview of the technological features associated with various pharmaceutical applications. Each app is uniquely identified by a serial number, and the table outlines its distinct technological capabilities, such as content distribution across platforms and secure login functionalities. This resource is valuable for users and developers, aiding in understanding the functionalities and potentials of these applications.

**Table 1:** Technological elements in mobile applications for online pharmacies in India.

**Serial no**	**Apps name**	**Specific origin or country of apps**	**Technical aspects of the app (all that apply)**
1	Netmeds	India	Allow sharing of different platforms
2	Genericart	India	Allow sharing of different platforms
3	Health mug	India	Allow sharing of different platforms
4	Schwabe	India	Allow sharing of different platforms
5	PositraRx -Online pharmacy	India	Allow sharing of different platforms
6	AyushCare.IN	India	Allow sharing of different platforms
7	Zenerics	India	Allow sharing of different platforms
8	Pulse pharmacy	India	Allow sharing of different platforms
9	FrankRosshealth	India	Allow sharing of different platforms
10	Tabletshablet	India	Allow sharing of different platforms
11	Indimedo	India	Allow sharing of different platforms
12	Hindustan pharmacy	India	Allow sharing of different platforms
13	Kerala pharmacy	India	Allow sharing of different platforms

Table 2 offers a concise summary of the primary focus areas, strategies, and affiliations of these apps. The analysis indicates that these applications aim to improve individual wellbeing while adopting a business-oriented approach through commercial partnerships. This information helps users comprehend the goals and target market of these applications. Table 3 provides a comprehensive analysis of pharmacy application manufacturers, including essential details such as evaluations, user feedback, the latest update date, and the app's pricing model (free or paid). This chart serves as a crucial tool for users to assess the reliability of these programs based on the creators' reputation and user feedback. Table 4 examines various versions of pharmaceutical software, including download statistics, release times, and compatible operating systems. This data is valuable for users seeking compatibility with their devices and the latest application versions to stay updated.

**Table 2:** Characteristics and objectives of digital pharmacy apps in India.

**Serial no**	**Apps name**	**Targeted objectives**	**Conceptual foundations**	**Associations**
1	Netmeds	Wellbeing	Business	Commercial
2	Genericart	Wellbeing	Business	Commercial
3	Health mug	Wellbeing	Business	Commercial
4	Schwabe	Wellbeing	Education	Unknown
5	PositraRx -Online pharmacy	Wellbeing	Business	Commercial
6	AyushCare.IN	Wellbeing	Business	Commercial
7	Zenerics	Wellbeing	Business	Commercial
8	Pulse pharmacy	Wellbeing	Business	Commercial
9	FrankRosshealth	Wellbeing	Business	Commercial
10	Tabletshablet	Wellbeing	Education	Unknown
11	Indimedo	Wellbeing	Business	Commercial
12	Hindustan pharmacy	Wellbeing	Business	Commercial
13	Kerala pharmacy	Wellbeing	Business	Commercial

**Table 3:** Information on the developer and distinct features of online pharmacy apps in India.

**Serial no**	**Apps name**	**Developers**	**No of reviews**	**Rating**	**Last update**	**Cost**
1	Netmeds	Netmeds marketplace limited	681	4.3	4 September 2023	Free
2	Genericart	Autoqed	Nil	Nil	12 September	Free
3	Health mug	Health mug	Nil	Nil	9 June 2023	Free
4	Schwabe	Dr. Willmar Schwabe India Pvt. Ltd	3k	3.8	9 august 2023	Free
5	PositraRx -Online pharmacy	Positra healthcare	Nil	Nil	8 September 2023	Free
6	AyushCare.IN	Ayush care	Nil	Nil	5 September 2023	Free
7	Zenerics	Zenerics	Nil	Nil	25 November 2023	Free
8	Pulse pharmacy	Pasumai pulse pharmacy	648	4.8	8 September 2023	Free
9	FrankRosshealth	Emami frank ross	Nil	Nil	21 September 2023	Free
10	Tabletshablet	Sterling Take Opinion pvt ltd	Nil	Nil	12 February 2023	Free
11	Indimedo	Indimedo.com	Nil	Nil	3 November 2022	Free
12	Hindustan pharmacy	Emedstore Pharma IT Company	Nil	Nil	20 December 2020	Free
13	Kerala pharmacy	Zibew E-commerce Services Pvt Ltd	Nil	Nil	9 March 2023	Free

**Table 4:** Application information encompassing version updates, download statistics, platform compatibility, and associated costs.

**Serial no**	**Apps name**	**Version**	**Downloads**	**Released on**	**Platform**	**Age**
1	Netmeds	8.2.64	10M+	8 November 2015	IOS	General
2	Genericart	1.0.47	100,000+	6 March 2021	Google play store	General
3	Health mug	8.5	500,000+	27 October	Google play store	General
4	Schwabe	2.94	100,000+	1 October 2015	Google play store	General
5	PositraRx -Online pharmacy	1.0.605	1,000+	4 January 2023	Google play store	General
6	AyushCare.IN	3.1.16	50,000+	23 March 2020	Google play store	General
7	Zenerics	1.0.1	1,000+	26 November	Google play store	General
8	Pulse pharmacy	2.2.16	50,000+	29 May 2019	Google play store	General
9	FrankRosshealth	1.0.67	100,000+	20 October 2021	Google play store	General
10	Tabletshablet	8.0.0	50,000+	9 January 2018	Google play store	General
11	Indimedo	1.8	10,000+	13 February 2021	Google play store	General
12	Hindustan pharmacy	1.0.8	5,000+	2 April 2019	Google play store	General
13	Kerala pharmacy	1.1	50+	9 March 2023	Google play store	General

**Figure 2:**
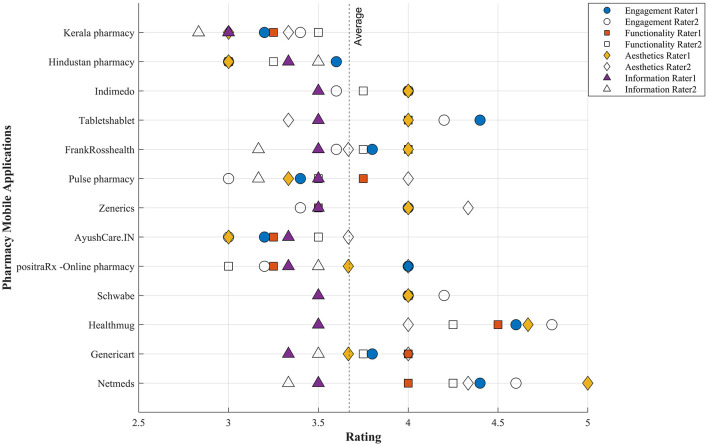
Average MARS scores for all applications, evaluated by two independent raters.

**Figure 3:**
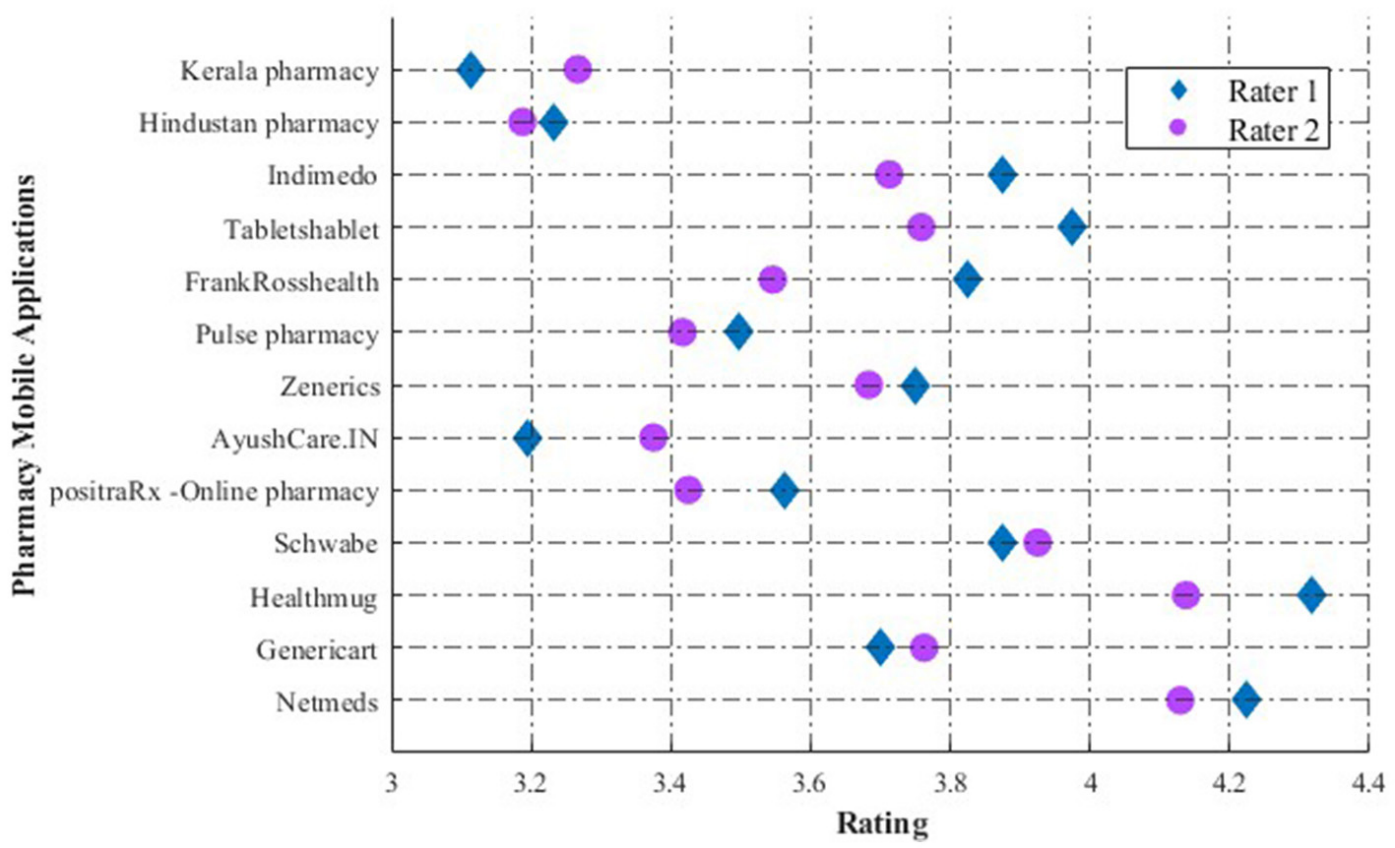
Mean scores for each MARS dimension across all applications, as evaluated by two raters.

Table 5 offers concise and informative descriptions of pharmacy applications, providing users with a summary of each app's features and unique value proposition. This feature is especially beneficial for individuals searching for an application that aligns with their healthcare and pharmaceutical needs. The average mean score of all applications by two raters using the MARS scale is depicted in Figure 2. The mean score of each of the dimensions of the MARS scale by two raters is illustrated in Figure 3. Table 6 displays the internal consistency and interrater reliability metrics for the MARS items and subscale scores. It also contains item-total correlations that have been rectified and descriptive data for individual items that come from independent assessments of 13 Indian online drugstore applications.

**Table 5:** Overview of digital pharmacy applications in India.

**Serial no**	**Apps name**	**About apps**
1	Net meds	Netmeds is a prominent internet-based pharmacy in India, that provides more reliable prescription, over-the-counter, and health items.
2	Genericart	Genericart, India's leading generic medicines provider, offers convenient online medicine ordering.
3	Health mug	Health Mug is a top healthcare app that provides a comprehensive solution for all medical, health, and personal care needs.
4	Schwabe	Schwabe is a renowned homeopathic medicine pharmacy.
5	Positra Rx -Online Pharmacy	Positra Rx offers an easy and convenient method to obtain medications as well as healthcare products.
6	AyushCare.IN	Ayush Care is India's digital ayurvedic store application, allowing users to place online orders for medicines.
7	Zenerics	Zenerics is a reliable online pharmacy in India that specializes in generic medicines.
8	Pulse pharmacy	Pulse pharmacy allows you to purchase Medications Online for Up to 20% Discount.
9	FrankRosshealth	Frank Ross Health is your ultimate destination for all your healthcare requirements.
10	Tabletshablet	Tabletshablet.com - Accessible Healthcare is an online platform for healthcare products.
11	Indimedo	Indimedo is a trusted online pharmacy app based in India.
12	Hindustan pharmacy	Hindustan pharmacy can access an extensive medicines database, encompassing over 55,000 medications.
13	Kerala pharmacy	Pharmacy App to easily order your medicines from any location within India.

**Table 6:** The interrater reliability and internal consistency metrics for both the MARS items and subscale scores.

**Subscale items**	**Item total correlation**	**Mean**	**Std dev**
**Engagement Cronbach alpha** = **0.85, ICC 0.748 (CI** = **0.358–0.916)**			
Entertainment	0.90	3.923	0.64051
Interest	0.67	3.7692	0.72501
Customization	0.82	4.0769	0.64051
Interactivity	0.67	3.6154	0.50637
Target group	0.36	3.7692	0.43853
**Functionality Cronbach alpha** **=** **0.88, ICC 0.794 (CI** = **0.453–0.932)**			
Performance	0.43	3.8462	0.55470
Ease of use	0.60	3.7692	0.59914
Navigation	0.63	3.5385	0.51887
Gestural design	0.22	3.7692	0.43853
**Aesthetic Cronbach alpha** **=** **0.63, ICC 0.464 (CI**= −**0.090** −**0.799)**			
Layout	0.72	3.8462	0.68874
Graphics	0.60	3.7692	0.59914
Visual appeal	0.49	3.7692	0.72501
**Information Cronbach alpha** = **0.83, ICC 0.713 (CI** = **0.292–0.903)**			
Accuracy of app description in the app store	0.43	3.9231	0.27735
Goals	0.43	3.9231	0.27735
Quality of info	0.36	4.0769	0.27735
Quantity of info	0.38	3.5385	0.51887
Visual info	0.40	1.3077	0.75107
Credibility	0.617	3.4615	0.51887
Evidence base	N/A	N/A	N/A
**Subjective quality Cronbach alpha** = **0.84, ICC** = **0.726 (CI**= **0.316–0.908)**			
Would you recommend this app to people who might benefit from it	0.61	3.1538	0.80064
How many times do you think you would use this app in the next 12 months if it was relevant to you?	0.51	3.6715	0.37036
Would you pay for this app?	0.33	1.3077	0.75105
What is your overall star rating for the app?	0.68	3.5133	0.48722

Additionally, it includes corrected item-total correlations and descriptive statistics for items, derived from the independent evaluations of 13 online pharmacy apps in India.

**Figure 4:**
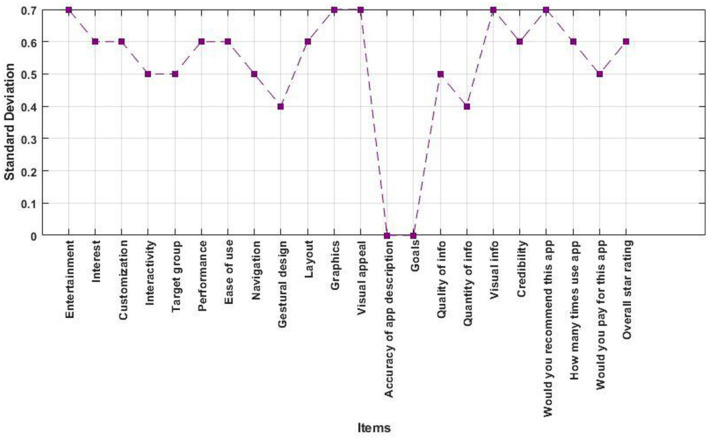
Variability of different items measured by standard deviation on the MARS Scale.

The survey findings provide a thorough evaluation of many aspects of user engagement and satisfaction with the app. The Engagement subscale, which has a great Cronbach alpha reliability of 0.85 and a considerable ICC of 0.748 (CI = 0.358–0.916), explores various dimensions like Entertainment, Interest, Customization, Interactivity, and Target Group. The mean ratings for Entertainment and Customization were notably high, with values of (3.923 and 4.0769), respectively. This indicates that users considered these features to be very engaging. On the other hand, the mean score for Target Group was lower at 3.7692, showing that there is space for development in targeting certain user groups. The standard deviation of the engagement dimension ranges between 0.43 and 0.72 demonstrated in Figure 4.

The Functionality subscale assesses Performance, Ease of Use, Navigation, and Gestural Design. It has a high level of internal consistency with a Cronbach alpha reliability of 0.88 and a strong test-retest reliability with an intraclass correlation coefficient (ICC) of 0.794 (CI = 0.453–0.932). While Performance and Ease of Use obtained mean ratings of 3.8462 and 3.7692 respectively, Navigation had a slightly lower mean of 3.5385, indicating possible usability difficulties. Gestural Design had the lowest mean of 3.7692, showing a need for improvement. The standard deviation of the functionality dimension ranges between 0.43 and 0.55 demonstrated in Figure 4.

The Aesthetic subscale examines Layout, Graphics, and Visual Appeal. It has a Cronbach alpha reliability of 0.63 and an ICC of 0.464 (CI = −0.090–0.799). The Layout category had the highest score, with a mean of 3.8462, followed by Graphics and Visual Appeal. This suggests an overall pleasant aesthetic experience. The standard deviation of aesthetic dimension ranges between 0.68 and 0.72 demonstrated in Figure 4.

The Information subscale evaluates many aspects such as the Accuracy of App Description, Goals, Quality of Info, Quantity of Info, Visual Info, Credibility, and Evidence Base (unavailable). It has a high Cronbach alpha reliability of 0.83 and an ICC of 0.713 (CI = 0.292–0.903). The measure of credibility was notably strong, with a mean value of 3.4615. On the other hand, the mean value for Visual Info was much lower at 1.3077, suggesting possible concerns with the presentation of visual information. The standard deviation of the information dimension varies from 0.27 to 0.75 shown in Figure 4.

The Subjective Quality subscale, with a Cronbach alpha reliability of 0.84 and an ICC of 0.726 (CI = 0.316–0.908), investigates users' subjective evaluations. Users showed a clear inclination to suggest the app (mean of 3.1538). However, their readiness to pay for the app resulted in a lower mean of 1.3077. The standard deviation of the subjective quality dimension varies from 0.37 to 0.80 shown in Figure 4.

The item-total correlation for the engagement dimension subsection was excellent (0.90) in the entertainment category, while it was satisfactory (0.70) in the target group. The performance of the functionality portion has a robust correlation (0.43), although the correlation with gestural design is minimal (0.22). The aesthetics portion arrangement correlates (0.72), whilst visual appeal shows a correlation of (0.49). The accuracy of the information part of the app exhibits a significant relationship of (0.43), while the quality of the information has a modest association of (0.36). The overall rating score of MARS of all applications of online pharmacies of India demonstrated good reliability by employing the ICC (0.464–0.794) and the total scores of apps showed great consistency by using Cronbach alpha (0.88). The average star rating of 3.5133 indicates that users typically have a positive impression. The comprehensive analysis provides developers with useful insights, identifying strong points and possible areas for improvement in the app's engagement, functionality, aesthetics, information provided, and overall subjective quality.

## 5 Discussion

This is the first study to assess the quality of Indian online applications. The objective of the research was to assess the quality of online pharmacy applications in India that are accessible through the Google Play Store and App Store, employing the Mars Scale. The Mars scale is an innovative, multidimensional (engagement, functionality, aesthetic, information, and subjective) evaluation framework designed specifically for mobile health applications (13). A total of forty applications were initially selected for this study from both app stores; however, following the application of inclusion and exclusion criteria, only 13 applications met the inclusion criteria. All applications were selected at random and were written in English. Many apps have high star ratings, usually ranging from 4.3 to 4.8 out of 5, which suggests that users are generally satisfied, according to an analysis of the app market. But, since these ratings may not always correctly reflect app quality, it's important to approach them cautiously. To gain more insight, this study used the MARS. An overall respectable level was indicated by the 3.11 out of 5 MARS score that the chosen applications combined to receive. It is noteworthy, however, that significant variation was found in several domains, most notably aesthetics, where scores varied from 3.0 to 5.0. This unevenness suggests that there is a wide range of app quality available right now, with certain examples pointing to the existence of lower-quality options.

The functionality subscale, which assessed the ease of use, performance, navigation, and gesture capabilities of applications, achieved the highest ICC value. Conversely, the aesthetic subscale, which evaluated the visual information, layout, and graphics of applications, received the lowest ICC value. As cited in previous research, the subjective component was omitted from the final Mars score. Our research demonstrated a lower ICC value in comparison to the previous study (26).

The majority of the applications were readily downloadable from the App Store and Android Market. Numerous applications exhibited authentication and security functionality issues. We limited our selection for this study to applications that offered simple authentication procedures, requiring neither a login name nor a mobile number. The information section of the majority of applications comprises fundamental medicine-related details, such as the dosage form, dose, indications, adverse effects, warnings, brand names, and cost of the medication. Traditionally, physicians were responsible for providing patients with information about their illness, its development, and potential treatments. However, with the rise of the internet, the dynamic between doctors and patients has shifted. Increasingly, individuals are turning to the Internet for self-diagnosis and treatment options. This trend toward self-medication is particularly significant in countries like India, where access to healthcare services may be limited for many people (27). In the 1990s, internet usage accounted for just 1% of the global population, but today, that figure has skyrocketed to 90% worldwide (28). By the end of 2019, India's internet users are projected to reach 627 million, representing 40% of the total population, with a significant portion utilizing the internet for self-medication and accessing health-related information. Self-medication is a global phenomenon, with prevalence ranging from 53% to 75% across different countries. Over the last decade, the prevalence of self-medication in India has surged from 31% to 71% (29).

Online pharmacy apps offer a range of services beyond just dispensing medications, including prescription refills and access to comprehensive drug information. Despite the central role pharmacies play in healthcare, there is a notable absence of studies exploring this aspect. Our study addresses this gap by examining the fundamental involvement, functions, layout, and information provided by pharmacy services within these apps.

### 5.1 Limitation

This research endeavor is not without its inherent limitations. The extent of our research was primarily restricted to a predetermined geographical area, specifically India, and specifically concentrated on applications that are available in the English language. The narrow scope used in this analysis may have unintentionally overlooked a broader global perspective, thus potentially disregarding valuable information obtained from a wider array of applications. Furthermore, our analysis is limited by its exclusive emphasis on free apps. The decision's omission of commercial apps failed to acknowledge the possible valuable perspectives and data they may provide. We must recognize that our technique mostly focused on app stores and did not include a thorough examination of existing literature. Therefore, our analysis may not have thoroughly examined the whole spectrum of available material and academic research on this specific subject. Another limitation of our study was eradicating the user's perspective and based on the MARS scale objectives However, this constraint may be addressed in a future investigation. Users' perspective, which may have resulted in a more acceptable assessment of the application.

### 5.2 Future recommendation

To enhance the reliability of online pharmacy applications in India, it is recommended to implement stricter regulations and guidelines, conduct regular audits to evaluate the content of these applications, strengthen data security measures, and provide standardized and evidence-based medical information. Further inquiries may concentrate on examining client views and opinions on these apps, with a specific emphasis on the dynamics of user-platform interaction and the building of trust. It is also recommended that further research be done using multiple mobile apps, patient population, and limited resources.

### 5.3 Ethics approval

Ethical approval was obtained for this study from Jinnah University for Women with reference number JUW/IERB/PHARM-ERA-004/2023.

## 6 Conclusion

The MARS which was used to evaluate mobile pharmacy applications in India, has yielded important insights into the dependability and efficiency of these apps. The study's findings highlight how crucial it is to make sure that online pharmacy platforms are reliable and legitimate. This study boosts the confidence of the consumer of online pharmacy applications as well as promotes the growth and quality of online pharmacy applications.

## Data Availability

The raw data supporting the conclusions of this article will be made available by the authors, without undue reservation.
